# Amyloid Beta Pathology Exacerbates Weight Loss and Brain Cytokine Responses following Low-Dose Lipopolysaccharide in Aged Female Tg2576 Mice

**DOI:** 10.3390/ijms23042377

**Published:** 2022-02-21

**Authors:** Rachel C. Knopp, Kristen K. Baumann, Miranda L. Wilson, William A. Banks, Michelle A. Erickson

**Affiliations:** 1 Geriatrics Research Education and Clinical Center, Veterans Affairs Puget Sound Health Care System, Seattle, WA 98108, USA; rcknopp@uw.edu (R.C.K.); kristen.baumann@va.gov (K.K.B.); miranda.wilson@icahn.mssm.edu (M.L.W.); wabanks1@uw.edu (W.A.B.); 2 Division of Gerontology and Geriatric Medicine, Department of Medicine, University of Washington School of Medicine, Seattle, WA 98195, USA

**Keywords:** inflammation, Alzheimer’s disease, amyloid beta, serum amyloid A, sickness behavior, depressive-like behavior, cytokines, chemokines

## Abstract

Systemic inflammation has been implicated in the progression of Alzheimer’s disease (AD); however, less is understood about how existing AD pathology contributes to adverse outcomes following acute inflammatory insults. In the present study, our goal was to determine how AD-associated amyloid beta (Aβ) pathology influences the acute neuroinflammatory and behavioral responses to a moderate systemic inflammatory insult. We treated 16–18-month-old female Tg2576 (Tg) mice, which overproduce human Aβ and develop plaques, and age-matched wild-type (WT) littermate mice with an intraperitoneal injection of 0.33 mg/kg lipopolysaccharide (LPS) or saline. Mice were then evaluated over the next 28 h for sickness/depressive-like behaviors (food intake, weight loss, locomotion, and sucrose preference), systemic inflammation (serum amyloid A, SAA), blood-brain barrier (BBB) disruption, astrogliosis (glial fibrillary acidic protein/GFAP), Aβ, and cytokine levels in the brain. We found that LPS caused a larger reduction in body weight in Tg vs. WT mice, but that other behavioral responses to LPS did not differ by genotype. BBB disruption was not apparent in either genotype following LPS. Concentrations of the systemic inflammatory marker, SAA, in the blood and brain were significantly increased with LPS but did not significantly differ by genotype. GFAP was increased in Tg mice vs. WT but was not significantly affected by LPS in either genotype. Finally, LPS-induced increases of eight cytokines (IL-1β, IL-6, IL-12 (p40), IL-10, IL-17A, MIP-1α/CCL3, MIP-1β/CCL4, and RANTES/CCL5) were found to be significantly higher in Tg mice vs. WT. In summary, our data show that Aβ pathology exacerbates the neuroinflammatory response to LPS and identifies cytokines that are selectively regulated by Aβ. The association of worse neuroinflammation with greater weight loss in Tg mice suggests that Aβ pathology could contribute to poor outcomes following a systemic inflammatory insult.

## 1. Introduction

Alzheimer’s disease (AD) is the most common cause of dementia and is pathologically defined by the deposition of amyloid-β (Aβ) plaques and tau neurofibrillary tangles in the brain [1]. Aβ and tau are thought to contribute to neurodegeneration in AD, but other factors such as inflammation can accelerate AD onset and progression [2]. AD brains have increased levels of inflammatory cytokines and immune factors, as well as reactive astrocytes and microglia in regions of the brain with high AD pathology [3,4]. In addition, both neurodegeneration and normative aging have been associated with glia that are more reactive to inflammatory stimuli [5,6]. Aging and AD have also been associated with increased levels of pro-inflammatory cytokines and other inflammatory factors in blood [7,8]. Therefore, the reactive state of glia as well as peripheral immune responses may increase vulnerability of the aging/AD brain to adverse outcomes following systemic inflammatory insults.

Lipopolysaccharide (LPS) is a prototypic immune stimulus that causes systemic immune activation, including in the brain [9]. LPS does not cross the intact blood-brain barrier (BBB) [10], but exerts effects on the brain through a number of indirect routes involving interactions with the BBB [11], the peripheral nervous system [12], and sites lacking an intact BBB such as circumventricular organs [13]. When given intraperitoneally or intravenously, LPS induces a predictable pattern of cellular, biochemical, and behavioral responses that have been well-characterized in rodents and humans [14,15]. Acute inflammatory insults like LPS or other inflammatory stimuli induce behavioral responses that are thought to aid in fighting infection and limiting the spread of disease [16,17]. The behavioral responses to LPS that initially occur are termed sickness behaviors (SBs) and include malaise, fatigue, anorexia, apathy, and irritability [18]. In mice, SBs are easily monitored by evaluating locomotion, food intake, and weight loss, and these behaviors are regulated by pro-inflammatory cytokine signaling in the brain [19,20]. LPS also induces depressive-like behaviors (DBs), which resemble SBs [16] but can be temporally distinguished. For example, DBs in mice such as reduced sucrose preference and increased immobility under conditions of inescapable stress can persist after cytokine-dependent SBs resolve [19,20]. DBs rely, in part, on the activation of indoleamine 2,3-dioxygenase (IDO) [20]. Both aging and neurodegeneration can exacerbate and prolong SB and DB responses to LPS [21,22,23]. However, it is not clear whether the combination of aging and AD exacerbates the behavioral response to an inflammatory insult vs. aging alone.

LPS effects have been evaluated in mouse models of Aβ overexpression/plaque pathology [24], but these studies have mostly addressed how systemic inflammation affects the course of AD rather than how AD-associated pathology influences inflammatory and behavioral responses to LPS. Studies in humans have shown that inflammatory insults are associated with greater mortality and morbidity in subjects with AD vs. without [25,26,27]. A sublethal dose of LPS was shown to exacerbate depressive-like behavior, BBB disruption, and expression of interleukin-6 (IL-6) in brains of young APP23 transgenic mice vs. controls, although these mice did not yet have pre-existing plaques or gliosis [28]. Another study compared 6-month-old vs. 16-month-old wild-type (WT) and Tg2576 (Tg) mice, reflecting ages when plaques are absent/sparse vs. very abundant, and found that a 25 µg IV LPS dose caused a stronger induction of pro-inflammatory cytokines in brains of old Tg mice vs. old WT mice. When young Tg were evaluated vs. WT, the genotype differences in cytokine expression following LPS were attenuated [29]. These findings suggest that there is an interaction between age and Aβ levels that exacerbates neuroimmune responses to pro-inflammatory stimuli, which, in turn, may impact the severity of behavioral outcomes. However, both mouse studies used high doses of LPS, which can cause shock and some lethality [30,31,32].

The present study aimed to evaluate whether older mice with (Tg) or without (WT littermates) Aβ overproduction and plaque pathology exhibited differences in their behavioral and biochemical responses to a 0.33 mg/kg dose of LPS. This relatively low dose was chosen based on doses used previously to study SB responses in aged mice [21,22]. Our prior work in young mice indicated that this dose would not cause blood-brain barrier (BBB) disruption [33], which further distinguishes our study design from prior work that evaluated the BBB [28]. We measured the severity of SBs and DBs caused by LPS in each strain, as well as the accumulation of soluble and insoluble Aβ in the brain and gliosis. In addition, we measured levels of an acute phase protein, serum amyloid A (SAA), in the blood and brain. SAA is highly upregulated by the liver during an acute inflammatory insult, and the magnitude of increase indicates the severity of the systemic inflammatory response [34]. SAA can also cross the intact BBB, and it has been shown to contribute to plaque deposition and to regulate gliosis [35,36,37,38] and depressive-like behaviors [39]. Thus, we also evaluated concentrations of soluble and insoluble SAA in the brain. To characterize the neuroinflammatory response, we measured levels of 23 cytokines in the brain, including pro- and anti-inflammatory cytokines, as well as chemokines and growth factors. Because all parameters were evaluated in the same cohort of mice, our results provide a novel, holistic view of the effects of Aβ pathology on CNS, systemic, and behavioral responses to a systemic inflammatory insult.

## 2. Results

**Figure 1:**
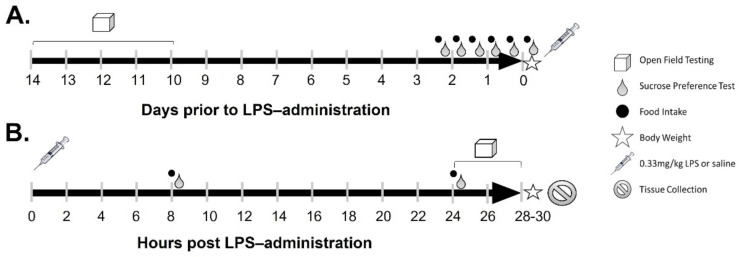
Schematic of the timing of behavioral tests and treatments. Pre–LPS treatment establishment of baselines in days is shown in (**A**) and timing of behavioral testing and tissue collection in hours post–LPS is shown in (**B**).

Female Tg mice and their WT female littermates, aged 16–18 months, were used for this study. Only female mice were chosen because they can be group-housed, whereas male Tg mice would have required single housing to avoid injuries from fighting, which could adversely affect some of the measured behavioral parameters. Female Tg mice exhibit worse Aβ-associated pathological changes and accelerated cognitive decline compared to their male counterparts [40], which is consistent with a more rapid AD progression observed in human females vs. males [41]. This study was conducted over 16 days (Figure 1), with behavior testing, sucrose preference, food intake, and body weights measured before and after treatment with low-dose LPS. Mice were sacrificed 28–30 h following acute LPS-injection.

### 2.1. Effects of LPS Treatment on SBs and DBs

**Figure 2:**
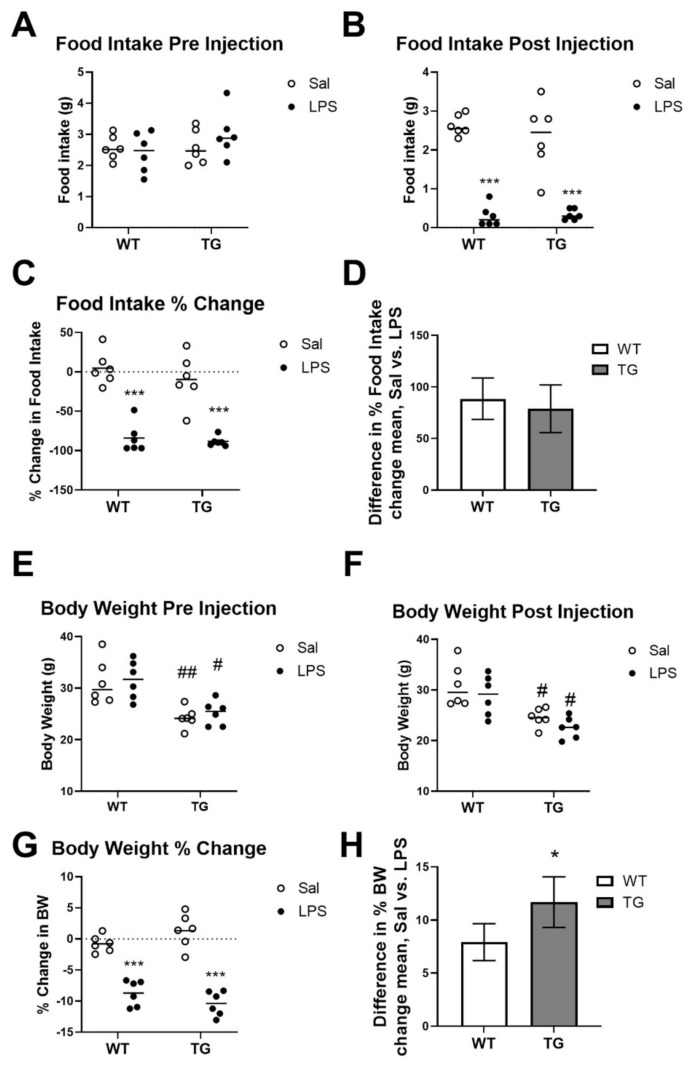
Effects of LPS on changes in food intake and body weight (BW) in wild-type (WT) and Tg2576 (TG) mice. Food intake before (**A**) and after (**B**) LPS injection. Changes in food intake between WT and TG (%) are shown in (**C**) and the difference between the means are shown in (**D**). Body weights before (**E**) and after (**F**) LPS injection. Changes in body weight between WT and TG (%) are shown in (**G**) and the difference between the means are shown in (**H**). Bars (**A**–**C**,**E**–**G**) indicate the mean, and error bars (**D**,**H**) are mean ± SD. * *p* < 0.05, *** *p* < 0.001 vs. saline within genotype. # *p* < 0.05, ## *p* < 0.01 vs. WT of the same treatment. *n* = 6 mice/group.

Inflammatory insults such as LPS are known to cause behavioral changes, which include reduced eating and drinking, weight loss, reduced locomotion, anhedonia, and changes in taste preference [16,19,20]. We first tested whether SBs differed in wild type vs. Tg mice in response to a low-dose of LPS. Pre-treatment differences and absolute changes in food intake and body weight post-treatment are shown in Figure 2. There were no significant differences among any groups in food intake pre-treatment (Figure 2A). There was a significant main effect of LPS treatment on the total (F (1, 20) = 114.5, *p* < 0.0001, Figure 2B) and percent change in food intake (F (1, 20) = 89.67, *p* < 0.0001, Figure 2C). Multiple comparisons testing showed that LPS treatment caused significant decreases in total (Figure 2B) and percent change (Figure 2C) food intake vs. saline (Sal) controls. There was no significant effect of genotype or interaction on the total or percent change in food intake, and the differences in the Sal and LPS means did not differ by genotype (Figure 2D), suggesting that LPS equally reduces food intake in WT and Tg mice. Tg mice had significantly lower pre-treatment body weights than the WT (F (1, 20) = 24.82, *p* < 0.0001), but there were no significant differences between treatment groups within the same genotype (Figure 2E). There was no significant main effect of LPS treatment on absolute body weight (Figure 2F), but there was a significant effect on the percent change in body weight (F (1, 20) = 132.5, *p* < 0.0001), and a significant interaction of genotype and treatment on body weight (F (1, 20) = 4.904, *p* = 0.0386), indicating that LPS-induced weight loss is slightly greater in Tg mice vs. WT (Figure 2G). Multiple comparisons testing showed that LPS caused significant decreases in body weight vs. saline in both genotypes. The difference in the Sal and LPS means was significantly higher in Tg mice vs. WT (Figure 2H).

**Figure 3:**
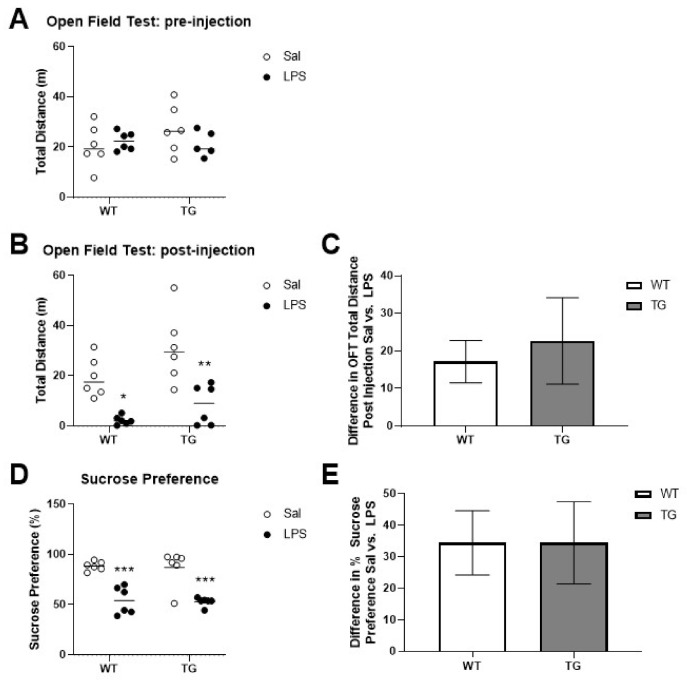
Effects of LPS on locomotor activity and sucrose preference. Total distance traveled in the open field test pre- (**A**) and post- (**B**) injection is shown as well as the difference between the means post-treatment in (**C**). Preference for sucrose is shown in (**D**) and the difference in % sucrose preference between the saline and LPS means is shown in (**E**). Bars indicate the mean, and the error bars indicate the SD. * *p* < 0.05, ** *p* < 0.01, *** *p* < 0.001 vs. saline within genotype. *n* = 6 mice per group.

There were no significant differences in pre-treatment open field activity among any groups (Figure 3A). Post-treatment (Figure 3B,C), there were significant main effects of treatment (F (1, 20) = 28.88, *p* < 0.001) and genotype (F (1, 20) = 5.875, *p* = 0.0250) on total distance travelled in the open field, which is a measure of locomotor activity. The increased activity in the Tg group was unexpected, but could reflect a greater stress response of Tg2576 mice vs. their WT counterparts to the IP injections [42]. Multiple comparisons testing showed that LPS caused significant reductions in locomotor activity vs. saline in both genotypes. Reduced sucrose preference is a depressive-like behavior that is caused by LPS treatment; however, this study design does not temporally resolve from symptoms of sickness. There was a significant main effect of treatment on sucrose preference (F (1, 20) = 51.61, *p* < 0.0001) and multiple comparisons tests showed that LPS caused significant reductions in sucrose preference vs. saline in both WT and Tg mice (Figure 3D), but the difference in Saline and LPS mean sucrose preference did not significantly change with genotype (Figure 3E).

### 2.2. Effects of LPS Treatment on BBB Disruption

**Figure 4:**
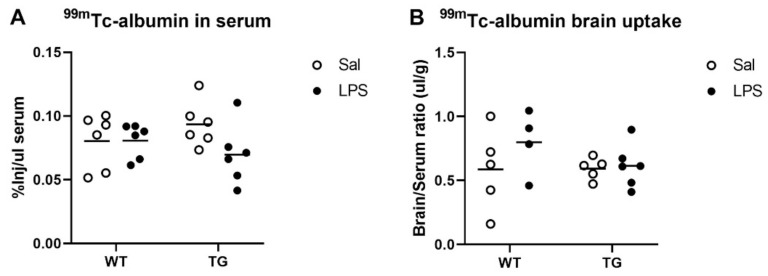
Effects of LPS on blood-brain barrier disruption. Circulating levels of ^99m^Tc-albumin are shown in (**A**), and brain/serum ratios of ^99m^Tc-albumin are shown in (**B**). Some samples (1 WT sal, 1 Tg sal, 2 WT LPS) were excluded from B due to incomplete brain vascular washout. *n* = 4–6 mice per group.

We predicted that the low LPS dose used in this study would not cause BBB disruption based on our prior work in young CD-1 mice [43]. To confirm this in the Tg and WT mice, we measured the leakage of ^99m^Tc-albumin injected into the jugular vein 28–30 h after LPS or saline treatment. The ^99m^Tc-albumin circulated for 15 min, and then arterial blood was collected, and the upper circulatory system was flushed with buffer to clear residual blood from the brain vasculature. ^99m^Tc-albumin was measured in serum and brains with a gamma counter. There were no significant effects of genotype or treatment on ^99m^Tc-albumin concentrations in the serum, indicating that the distribution volume of the albumin was similar in both groups (Figure 4A). Next, we analyzed the brain/serum ratios of ^99m^Tc-albumin, which is a measure of albumin that has leaked into the brain due to BBB disruption. There were no significant differences in the ^99m^Tc-albumin brain/serum ratios found due to genotype or treatment, indicating that BBB disruption did not occur as a result of either parameter (Figure 4B).

### 2.3. Effects of LPS Treatment on SAA Concentrations in Blood and Brain

**Figure 5:**
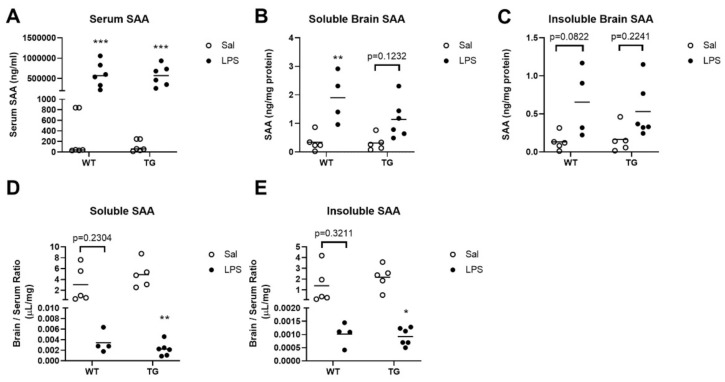
Effects of LPS on serum amyloid A levels in the blood and brain. Levels of serum SAA are shown in (**A**), and soluble and insoluble brain SAA levels are shown in (**B**,**C**), respectively. Brain/serum ratios of soluble and insoluble brain fractions are shown in (**D**,**E**), respectively. Some samples (1 WT sal, 1 TG Sal, 2 WT LPS) were excluded from (**B**–**E**) due to incomplete brain vascular washout, because this resulted in substantial SAA contamination from blood. * *p* <0.05, ** *p* < 0.01, *** *p* < 0.001 vs. saline treatment within genotype. *n* = 4–6 mice/group.

Next, we measured levels of SAA, an acute phase protein and sensitive marker of inflammation, in the blood and brain. LPS treatment significantly upregulated serum SAA levels in both genotypes (F (1, 20) = 50.27, *p* < 0.0001, Figure 5A). However, there was no significant difference between genotypes. LPS treatment also had a significant main effect on brain levels of soluble (F (1, 16) = 21.02, *p* = 0.0003, Figure 5B) and insoluble SAA (F (1, 16) = 10.74, *p* = 0.0047, Figure 5C). Multiple comparisons testing showed that in the WT mice, LPS significantly increased soluble SAA in brain vs. saline controls (*p* = 0.0048), but the effects of LPS on Tg mice were not significantly different from saline controls (*p* = 0.1232, Figure 5B). In the insoluble brain fractions, multiple comparisons testing did not detect any significant differences in saline vs. LPS treatments in either genotype. LPS treatment had an overall effect of lowering brain/serum ratios for soluble SAA (F (1, 16) = 18.18, *p* = 0.0006, Figure 5D) and insoluble SAA (F (1, 16) = 14.63, *p* = 0.0015, Figure 5E), and treatment means significantly differed only in the Tg group, although there was not a significant interaction of genotype and treatment for either brain/serum ratio calculated.

### 2.4. Effects of LPS Treatment on Aβ Concentrations in Brain

**Figure 6:**
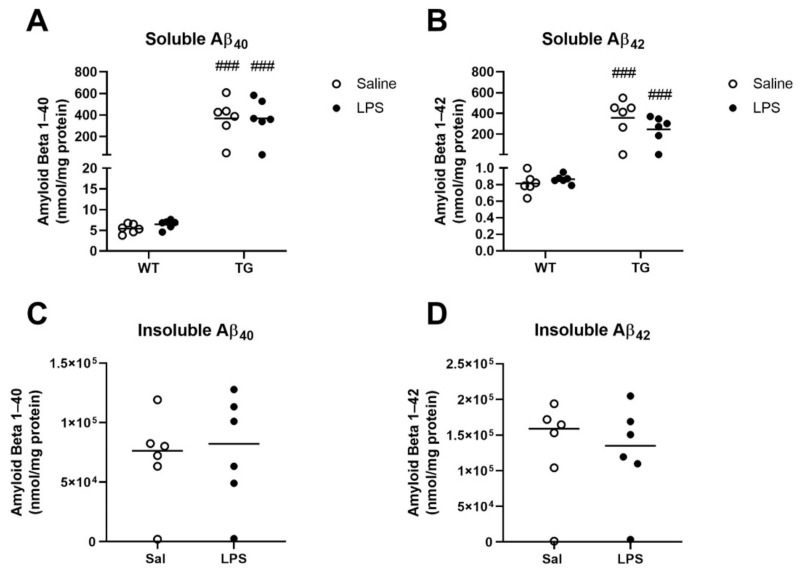
Effects of LPS on Aβ levels in brain. Soluble Aβ was measured in brains of WT and Tg mice (**A**,**B**), and insoluble Aβ was measured in Tg mice only (**C**,**D**). ### *p* < 0.001 vs. WT of the same treatment as analyzed by Tukey’s multiple comparisons post-test. *n* = 6 mice/group.

Next, we sought to understand if LPS affected the concentrations of Aβ in the brain acutely. To do so, we measured levels of Aβ_1–0_ and Aβ_1–42_ in the brains of WT and Tg mice by ELISA. We measured soluble Aβ in both genotypes but limited our measures of insoluble/aggregated Aβ to Tg mice because this pool of Aβ is not detectable in WT mice. Genotype had a significant effect on soluble Aβ_1–40_ (F (1, 20) = 43.68, *p* < 0.0001, Figure 6A) and Aβ_1–42_ (F (1, 20) = 37.99, *p* < 0.0001, Figure 6B), but there were no significant effect of LPS on soluble Aβ levels in the brain of either genotype. LPS had no significant effect on concentrations of insoluble Aβ in Tg mice (Figure 6C,D).

### 2.5. Effects of LPS on a Marker of Gliosis

**Figure 7:**
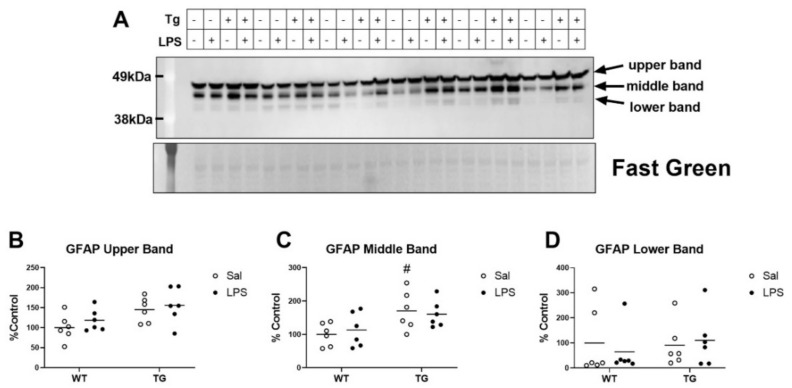
Effects of LPS on GFAP expression in brains of WT and Tg mice. Immunoblot shows three bands detected, reflecting different GFAP isoforms in the brain (**A**). Quantification of the immunoreactive signal normalized to fast green is shown for the upper (**B**), middle (**C**), and lower (**D**) bands. *n* = 6 mice per group. # *p* < 0.05 vs. WT of the same treatment.

Gliosis has been shown to increase in AD brains, and increases in glial reactivity could contribute to the heightened neuroinflammatory response and behavioral effects caused by systemic inflammatory stimuli [17]. We thus sought to quantify levels of glial fibrillary acidic protein (GFAP), a marker of reactive astrocytes, in brains of WT and Tg mice treated with LPS via immunoblot (Figure 7A). We detected three GFAP immunoreactive bands at sizes that are consistent with GFAP isoforms in the brain that have been described in WT and APP transgenic mice [44]. LPS did not have a significant effect on GFAP expression in either genotype (Figure 7B–D). However, genotype did have a significant effect on GFAP expression of the upper (F (1, 20) = 8.503, *p* = 0.0085, Figure 7B) and middle (F (1, 20) = 9.429, *p* = 0.0060, Figure 7C) GFAP bands, but not on the lower band. These data suggest that astrocytes are in a more reactive state in Tg mice vs. WT, but astrogliosis is not affected by this low dose of LPS at the timepoint studied.

### 2.6. Effect of LPS on Inflammatory Brain Cytokine Levels

**Figure 8:**
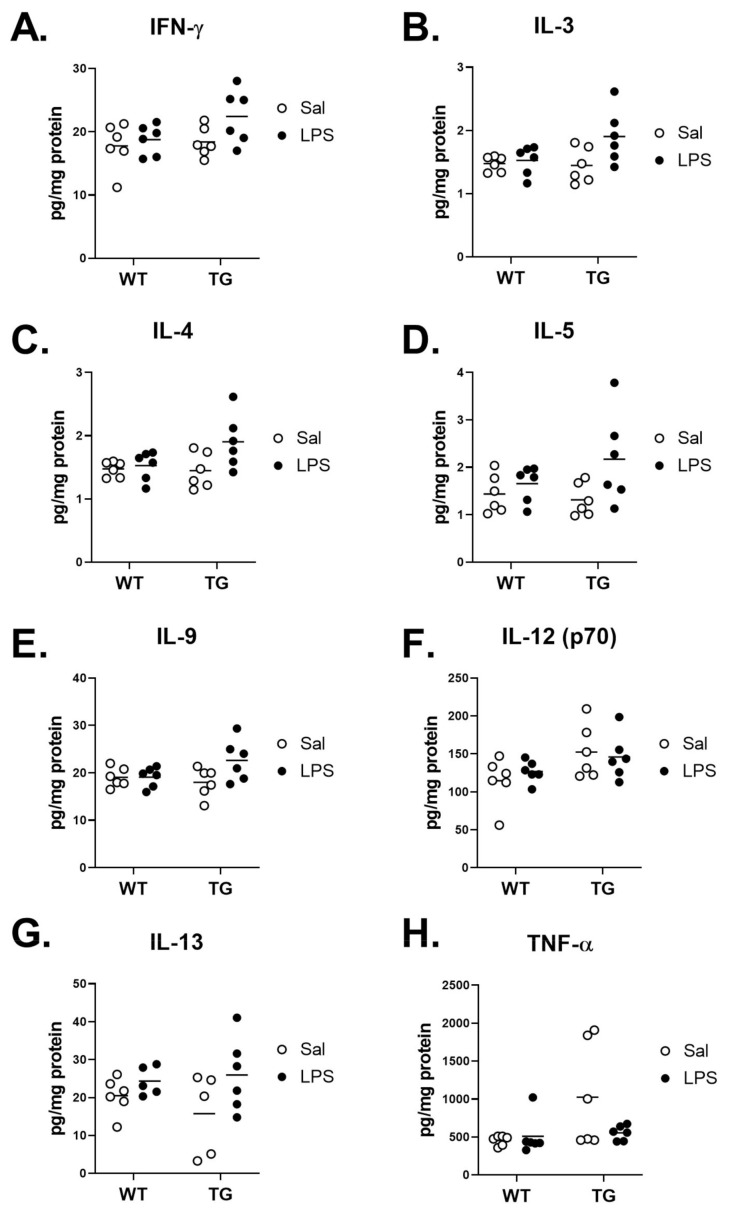
Cytokines with no significant mean differences of treatment within genotype or genotype within treatment. Cytokines IFN-γ (**A**), IL-3 (**B**), IL-4 (**C**), IL-5 (**D**), IL-9 (**E**), IL-12 (p70, (**F**)), IL-13 (**G**), and TNF-α (**H**) are shown and analyzed by Tukey’s multiple comparisons post-test. *n* = 6 mice/group.

We further sought to characterize cytokine responses in the brains of WT and Tg mice post-LPS by measuring the protein concentrations of cytokines in the brain. Table 1 summarizes the main effects of genotype and treatment and the interactions from Two-way ANOVA analysis. Of all cytokines analyzed, 4 displayed some readings that were either extrapolated or not detected and assigned a concentration of zero, and these are detailed in Table S2. No significant effects of genotype or treatment were found for IFN-γ, IL-9, or TNF-α. Significant effects on cytokines could generally be categorized into three groups: those in which there was an effect of genotype but not LPS treatment (IL-12 (p70)), those in which there was a significant effect of LPS treatment without a genotype interaction (CCL11, G-CSF, IL-2, IL-3, IL-4, IL-5, IL-13, CXCL1, and CCL2), and those in which there was a genotype x treatment interaction (IL-17A, GM-CSF, IL-1α, IL-1β, IL-6, IL-10, IL-12 (p40), CCL3, CCL4, and CCL5). We then further evaluated differences in cytokine expression by comparing the mean differences in expression levels within treatment and genotype. Interestingly, there were no apparent differences in the cytokine levels of WT vs. Tg saline groups for any cytokine that was measured. Figure 8 depicts the cytokines that did not significantly differ by treatment within genotype or by genotype within either treatment. These cytokines were both pro-inflammatory (interferon gamma (IFN-γ), Interleukin (IL)-12 (p70), tumor necrosis factor α (TNF-α)), and anti-inflammatory (IL-3, IL-4, IL-5, IL-9, IL-13).

**Figure 9:**
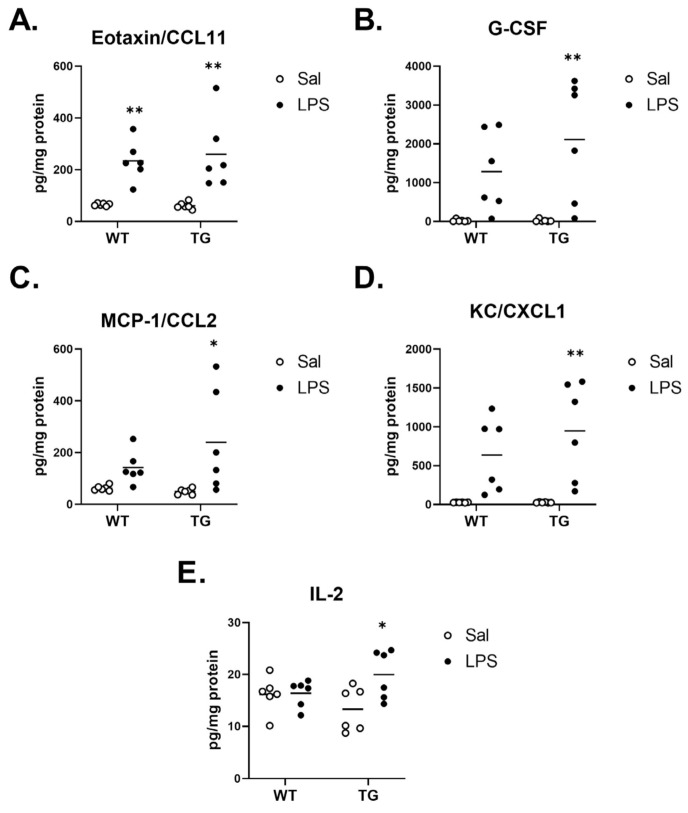
Cytokines with a significant mean difference of treatment within genotype (WT and/or Tg), without a significant treatment x genotype interaction. Cytokines Eotaxin/CCL11 (**A**), G-CSF (**B**), MCP-1/CCL2 (**C**), KC/CXCL1 (**D**), and IL-2 (**E**) are shown. * *p* < 0.01, ** *p* < 0.001 vs. saline treatment within genotype as determined by Tukey’s multiple comparisons post-test. *n* = 6 mice/group.

Figure 9 shows the cytokines that were significantly elevated in WT and/or Tg mice, but did not show a significant treatment x genotype interaction. Concentrations of the chemokine eotaxin/CCL11 significantly increased in both genotypes following LPS (Figure 9A). Levels of granulocyte colony-stimulating factor (G-CSF), the chemokines monocyte chemoattractant protein-1 (MCP-1)/CCL2, keratinocyte-derived chemokine (KC)/CXCL1, and the pro-inflammatory cytokine IL-2 significantly increased in the Tg mouse brains, but not in the WT (Figure 9B–E, respectively).

**Figure 10:**
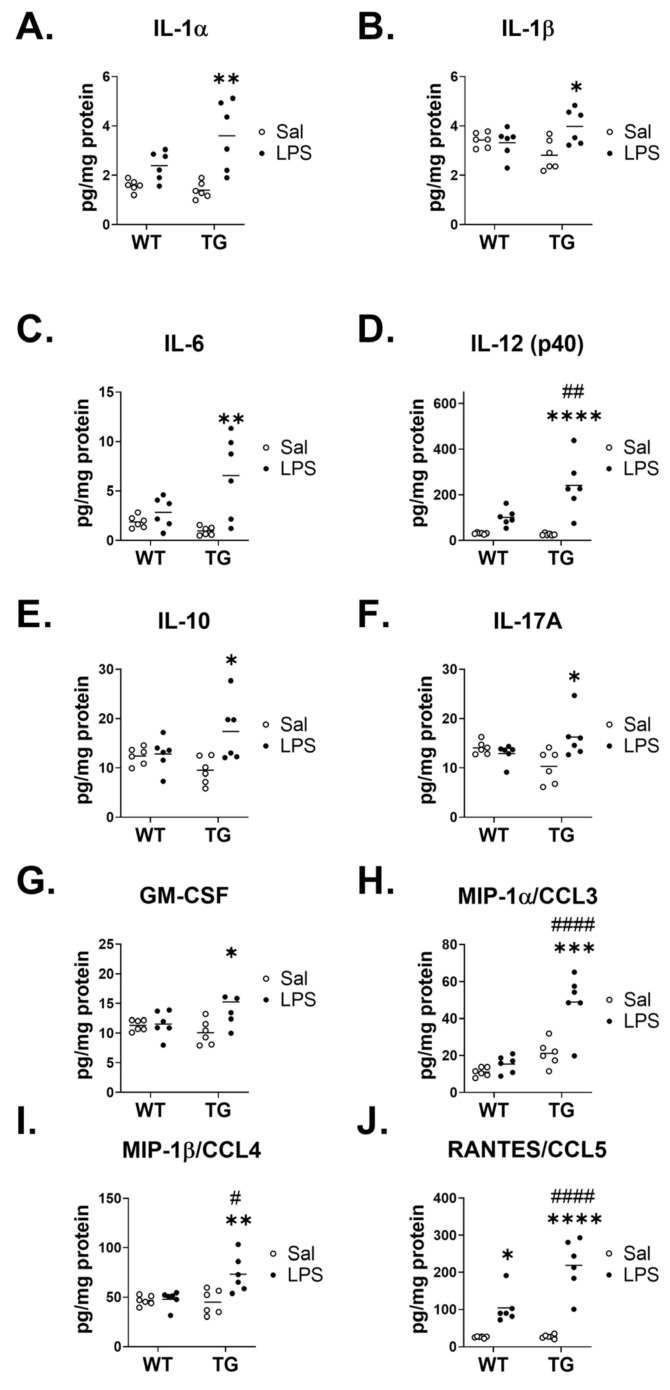
Cytokines showing a significant mean difference of treatment within genotype (WT and/or Tg), with a significant treatment x genotype interaction. IL-1α (**A**), IL-1β (**B**), IL-6 (**C**), IL-12 (p40, (**D**)), IL-10 (**E**), IL-17A (**F**), GM-CSF (**G**), MIP-1α/CCL3 (**H**), MIP-1β/CCL4 (**I**), and RANTES/CCL5 (**J**) are shown. * *p* < 0.05, ** *p* < 0.01, *** *p* < 0.001, **** *p* < 0.0001 vs. saline within genotype. # *p* < 0.05, ## *p* < 0.01, #### *p* <0.0001vs. WT of the same treatment, as determined by Tukey’s multiple comparison’s post-test. *n* = 6 mice/group.

Figure 10 shows the cytokines that had significant treatment x genotype interaction terms, which included proinflammatory cytokines (IL-1α, IL-1β, IL-6, and IL-12 (p40)), anti-inflammatory cytokines (IL-10, IL-17A), growth factors (granulocyte macrophage-colony stimulation factor (GM-CSF)), and chemokines (macrophage inflammatory protein (MIP)-1 α/CCL3, MIP-1 β/CCL4, and regulation on activation, normal T cell expressed, and secreted (RANTES)/CCL5). The mean concentrations of each cytokine in this group were generally elevated to a greater extent by LPS in the Tg group vs. WT, and this is further supported by Table 2, which shows that the mean differences in the cytokine expression of LPS and saline groups were significantly higher in the Tg mice and statistically differed from WT except for IL-1α and GM-CSF, which showed a trend.

We further analyzed the groups with a significant interaction to determine whether the difference in LPS and saline means differed across genotype (Table 2). Of all the eight cytokines, IL-1β, IL-6 IL-12 (p40), IL-10, IL-17A, MIP-1α/CCL3, MIP-1β/CCL4, and RANTES/CCL5 had a significant difference between means. These data suggest that amyloid beta pathology exacerbated the response to these eight cytokines following LPS stimulus.

## 3. Discussion

Both aging and AD-associated plaque pathology can increase the neuroinflammatory response to systemic inflammatory insults in mice [21,29]. However, it has not been evaluated whether Aβ pathology plus aging could cause more pronounced neuroinflammatory and behavioral responses to an insult than aging alone. Prior work showed that aged Tg mice had increased cytokine upregulation in the brain in response to LPS vs. aged WT [29], and enhanced brain cytokine responses have been associated with exacerbated SBs and DBs in aged mice [21,22]. Therefore, we reasoned that Tg mice would have increased glial reactivity and cytokine accumulation vs. WT and would thus mount stronger behavioral responses to LPS, which may be associated with changes in brain levels of Aβ and/or SAA. We did find that GFAP expression was increased in brains of Tg mice, indicating that there was increased astrogliosis, which has been shown before [29]. However, we did not find any genotype differences in the baseline expression of cytokines. LPS induced pronounced SBs and DBs in both WT and Tg mice, and our results suggested that the effect of LPS on weight loss was significantly greater in Tg mice vs. WT. However, there was no significant genotype × treatment interaction for food intake, which could suggest differences in metabolic responses to LPS between strains. Tg mice were more active overall in the open field following treatments, and we cannot rule out that increased activity could also have contributed to more weight loss. However, weight loss is also a predictor of poor clinical outcomes in hospitalized elderly patients with infections [45,46], suggesting that there could be a clinically relevant impact of amyloid pathology on the response to inflammation.

Despite overall differences in GFAP protein levels in brains of WT vs. Tg mice, LPS had no significant effect on GFAP expression at the time point studied in either strain. The isoforms detected are consistent with isoforms described in WT and APP transgenic mice [44]. These splice variants are differentially expressed in the mouse brain, and some are associated with certain subtypes of astrocytes. However, our study was limited in identifying the specific isoforms expressed due to the broad specificity of the GFAP antibody used.

LPS also did not change Aβ levels in the brains of WT or LPS mice, which has been shown before to peak at earlier time points and return to baseline by 18 h [29]. LPS did increase concentrations of SAA in the blood and brain of both WT and Tg mice; however, there was not a significant genotype difference regarding baseline or LPS-induced SAA levels. Prior works have shown that SAA accumulates in the brain and is associated with plaques in AD [35]. In mice and humans, two isoforms of SAA, SAA1 and SAA2, are overexpressed by the liver and secreted into the bloodstream in response to inflammatory insults [47]. SAA1 and SAA2 can cross the intact mouse BBB [38]. Mice have a third isoform, SAA3, which is also induced by inflammation in tissues other than the liver but has a relatively low contribution to the total circulating SAA pool [48]. It has been shown that SAA1 overexpression in APP transgenic mice promotes the accumulation of Aβ in the brain and increases gliosis [36]. Conversely, APP overexpression was also found to increase SAA1 and SAA3 deposition in the brain [36,37]. However, SAA3 was shown to inhibit astrogliosis in Aβ-overproducing APP/PS1 mice, indicating that the functions of SAA in the brain may differ by isoform and depend on the conditions of expression. Our data indicate that under conditions of acute endogenous SAA overexpression due to inflammation, the overexpression and deposition of human Aβ in the brain does not affect the accumulation of SAA in the brain. However, our data do not rule out the possibility that an assessment of more chronic or prolonged responses to LPS would reveal an Aβ effect. LPS significantly lowered SAA brain/serum ratios for both soluble and insoluble SAA forms. Brain uptake of proteins that are transported across the BBB can depend on the concentration of that protein in blood. The substantial reductions in brain/serum ratios with LPS indicate that only a small portion of the upregulated circulating SAA gets into the brain. This may be due to saturation of the SAA transport system, or because only a portion of circulating SAA is available for transport. The majority of SAA in blood circulates bound to HDL, which does not cross the BBB, and only a very small portion of SAA circulates in a lipid-free form [49]. Therefore, the reduced brain/serum ratios with LPS may reflect the large pool of increased circulating SAA that is not accessible to transport across the BBB because of binding to HDL.

Cytokines are small cell-signaling molecules that are released following inflammatory stimuli and are crucial in modulating inflammatory responses. In our studies, there were no observed differences in baseline expression of cytokines between WT and Tg mice. These results agree with our previous study looking at 13-month-old male Tg2576 mice vs. WT [50]. However, another group has shown that brain concentrations of IL-1α, IL-1β, TNF-α, and MCP-1 are increased in 16-month-old Tg2576 mice compared to WT [29], although this study did not indicate the sex of the mice used.

In the panel of 23 cytokines, we detected 10 cytokines and chemokines that had a significant interaction in genotype x treatment. Of these, IL-1β, IL-6, IL-12 (p40), IL-10, IL-17A, MIP-1α/CCL3, MIPβ/CCL4, and RANTES/CCL5 were upregulated by LPS to a greater extent in Tg3576 mice vs. wildtype. Thus, we can conclude that the underlying amyloid pathology mediates their exacerbated response to acute LPS. This agrees with previous literature, which found elevated IL-1β in response to LPS in Tg2576 mice vs. WT [29], and ameliorated IL-1β levels in aged Tg2576 mice following antisense oligonucleotide treatment against AβPP [50]. IL-1 (a family of interleukins that includes IL-1α and IL-1β) has been characterized as a critical mediator of sickness behavior [51], as has IL-6 and TNF-α [52]. Intriguingly, our studies found no change in TNF-α levels in response to strain or LPS. In general, a cytokine network functions by the combined activity of all cytokines; that is, a cytokine never acts alone, but relies on others. However, our study identifies for the first time a unique expression pattern of cytokines in the brain that are differently regulated by amyloid pathology in the context of inflammation. Our study also offers novel insight on how cytokine profiles relate to behavioral outcomes, showing that weight loss, but not other SBs or DBs, is associated with the exacerbated neuroinflammatory response to LPS.

Our work does include some limitations that require further consideration. First, we limited our studies to female mice, whereas much of the literature has evaluated male mice. We reasoned that female mice would be appropriate to test our research question because female Tg develop worse AD pathology and cognitive changes vs. males with age [40]. Thus, if Aβ pathology was contributing to vulnerability to LPS, females would be expected to have worse outcomes. Females also have more microglial activation with age vs. males [53], suggesting that age-associated microglial priming could be more pronounced in female mice.

Second, we did not compare our aged mice to younger mice and so did not assess the effects of aging alone. However, age-dependent differences in neuroinflammatory responses of Tg mice to LPS have been reported elsewhere [29], and our goal here was to further understand how Aβ pathology in the context of age influences responses to LPS. In the commonly used aging mouse model, C57BL/6J, old age is usually categorized as 18–24 months, but survivorship at 24 months is above 75%. Survivorship in this study was 60% in both Tg mice and their WT counterparts at 16–18 months, whereas a 60% survivorship at 22 months was reported in male Tg mice [54].

Third, our study was limited to a single dose of LPS and a single time point for studying behavioral parameters. We chose a single low-dose of LPS since it had been well-characterized in terms of behavioral responses [21,22]. We also wanted to choose a dose that would not cause BBB disruption so that we could interpret SAA accumulation in the brain that would be attributable to transport across the BBB rather than a combination of transport and BBB leakage. Despite the lack of differences found between WT and Tg mice with LPS in our study, another group has shown that there are worse neuroinflammatory outcomes, BBB disruption, and SBs in young APP transgenic mice that have not yet developed plaques vs. WT treated with a 10 mg/kg dose of LPS [28]. This dose is just below the threshold of causing lethality for young mice [31], which could indicate that Aβ accumulation in the brain has greater influence when the inflammatory insult is severe. Although the study did not consider aging, the results are consistent with findings in humans that patients with AD are more likely to die of infections than age-matched patients without dementia [26]. Other groups have found that 24 h was an optimal time point to detect the persistence of SBs and DBs in young vs. aged mice at the LPS dose used in this study [21,22]. Further, behavioral responses to low-dose LPS tend to be the worse 6–8 h after a single LPS injection and begin to improve around 24 h in aged mice [21], which corresponds with cytokine expression patterns in the brains of aged WT and Tg mice [29]. However, we did not evaluate the time to recovery, which could differ and, thus, would be an important future direction.

One final important consideration of our work is that Tg mice do not develop the full spectrum of AD pathology. Tg mice lack neurofibrillary tangle deposition and do not show much evidence of neurodegeneration, despite an abundance of CNS Aβ. Therefore, it may be that vulnerability to inflammation in AD would be enhanced by neuropathological features other than Aβ overexpression/deposition. In conclusion, our work indicates that Aβ pathology in aged mice increases weight loss and brain cytokines in response to LPS but does not modify effects of LPS on other SBs and DBs, SAA levels in blood or brain, astrogliosis, BBB disruption, or Aβ levels in the brain. Our results suggest that the responding cytokine network may contribute to adverse outcomes in AD patients exposed to inflammatory insults [26,55].

## 4. Materials and Methods

### 4.1. Mice

All mice were treated in accordance with NIH Guidelines for the Care and Use of Laboratory Animals in an AAALAC-accredited facility and approved by the Institutional Animal Care and Use Committee of the VA Puget Sound Health Care System (Protocol number 0934). Female Tg2576 mice (stock no. 1349-RD1-F) and their wild-type female littermates were purchased from Taconic at 12 weeks of age and housed at the VA Puget Sound Health Care System until they reached 16–18 months of age, at which point they were studied. Mice were kept on a 12/12-h light/dark cycle with ad libitum food (LabDiets cat no 5053) and water. A total of 19 wild-type and 20 Tg2576 mice were allocated for this study, and 12 from each group survived to endpoint. Mice that did not survive were either found dead in their cages or were euthanized for humane reasons, which included development of rapid weight loss, bloating/edema, tremors, and/or persistent lethargy. More information on the non-survivor mice is provided in Table S1.

### 4.2. LPS Treatments

Lipopolysaccharide from S. typhimurium (Sigma Aldrich cat no. L6511, St. Louis, MO, USA) was prepared in sterile normal saline and filter sterilized in a 0.22 µM filter prior to injection. Mice were injected i.p. with 0.33 mg/kg LPS, a dose that induces inflammatory responses and SBs in mice but does not cause BBB disruption [21,22,33]. Control mice were injected with normal saline. Injections occurred at 8 a.m. (08:00), and Figure 1 depicts the timing of treatments as they relate to measurements of behaviors and biochemical changes, which are described below.

### 4.3. Calculation of Food Intake and Body Weight

Overnight food intake in single-housed mice was determined by weighing food at 4 p.m. (16:00) and again at 8 a.m. (08:00) for three nights prior to injections, and then the night after treatment. The percent change in overnight food intake following injections of saline or LPS was determined by subtracting the overnight 3-day average food intake (baseline) from the overnight food-intake post-treatment, dividing by the baseline, and multiplying by 100%. The percentage change in body weight was determined by subtracting the body weight measured pre-injection from the body weight measured 28 h following injection.

### 4.4. Open Field Testing

Open field testing was conducted 10–14 days prior to LPS injections to establish a baseline, and then again 26–28 h after LPS or saline injections, between the hours of 10 am and 12 pm (10:00–12:00). Mice were placed in the testing room with dim light (8–10 lux) and allowed to adapt for 1.5 h. They were then placed into the center of a square open field with dimensions 40 × 40 cm and activity in the field was recorded for 10 min using ANY-maze video tracking software (Stoelting Co. Wood Dale, IL, USA). The center of the field was defined as a square with dimensions 20 × 20 cm.

### 4.5. Sucrose Preference Testing

Mice were single-housed and adapted to drinking water from two sipper bottles filled with water for 3 days. The morning after habituation, one of the water bottles was replaced with a 3% sucrose solution, which was determined to be the optimal concentration of sucrose to achieve at least 80% preference. Overnight liquid consumption was then evaluated by weighing the bottles at 4 p.m. (16:00) and again at 8 a.m. (08:00). Baseline sucrose consumption was recorded for three nights, and each morning the bottles were switched in their left/right orientation. Sucrose preference testing post-treatment commenced at 16:00 the day of treatment and bottles were re-weighed at 08:00 the next day.

### 4.6. Labeling of Albumin with ^99m^Tc-Pertechnetate

Bovine serum albumin (Sigma, St. Louis, MO, USA) was labeled with ^99m^Tc-pertechnetate (GE Healthcare, Seattle, WA, USA) using the stannous tartrate method [56] with modifications. Briefly, 120 µg of stannous tartrate (MP Biomedicals, Santa Ana, CA) and 1 mg bovine serum albumin was added to 500 µL of deionized water and dissolved. Hydrochloric acid was added to the tube to adjust the pH to 2.5–3.3. A measure of 1 mCi of ^99m^Tc-pertechnetate was added to the reaction tube and incubated for 20 min. The labeled bovine serum albumin (^99m^Tc-albumin) was then purified on a Sephadex G-10 column and the fractions with the highest activity were reserved. A measure of 1 µL of the reserved fractions was added to a 1% unlabeled bovine serum albumin solution and precipitated in a final volume of 15% trichloroacetic acid. The samples were centrifuged at 4200× *g* for 5 min, and then the pellets and supernatants were separated and counted in a Wizard 2 gamma counter (Perkin Elmer, Waltham, MA, USA). The % activity in the pellet reflects intact ^99m^Tc-albumin and was always greater than 90%. ^99m^Tc-albumin was used for experiments on the same day of labeling.

### 4.7. Evaluation of Blood-Brain Barrier Disruption

Evaluation of BBB disruption was conducted according to a previously described protocol with minor modifications [43]. Mice were anesthetized with i.p. urethane, 28–30 h after LPS or saline injections, and injected in the jugular vein with 5 million CPM of ^99m^Tc-albumin diluted in 1% bovine serum albumin (BSA) in lactated Ringer’s solution. After a circulation time of 15 min, arterial blood was collected from the abdominal aorta. The vascular space of the brain was washed free of blood by opening the thorax, clamping the descending thoracic aorta, severing both jugular veins, and perfusing 20 mL of lactated Ringer’s solution through the left ventricle of the heart in about 1.5 min. After washout, the mouse was immediately decapitated and the whole brain was removed, cut along the sagittal suture, and the hemispheres were weighed and visually inspected for evidence of incomplete washout. Four brains (1 WT sal, 1 Tg sal, 2 WT LPS) were excluded from analysis of BBB disruption and SAA concentrations in brain due to incomplete washout, which would interfere with measurements due to blood contamination in brain tissue. We did not exclude partially washed-out brains from Aβ or GFAP measurements since residual blood would contain little to no GFAP or Aβ compared to brain. For all brains, one hemisphere was counted in a Wizard2 2470 automatic gamma counter (Perkin Elmer, Waltham, MA, USA), and the other was frozen in liquid nitrogen for protein extraction. Serum was obtained by centrifuging the blood for 10 min at 2,500× *g* at 4 °C and transferring to a clean tube. Two µL serum was counted on a gamma counter, and the remainder was aliquoted and frozen on dry ice. Frozen samples were stored at −80 °C for 1 week prior to subsequent processing and analysis to allow for full decay of ^99m^Tc. ^99m^Tc albumin CPM in brains were first expressed as a percent of total injected material (%Inj) and then divided by brain weight (%Inj/g). Next, the concentration of ^99m^Tc-albumin in serum was calculated as %Inj/µL. Finally, the brain/serum ratios of ^99m^Tc-albumin were calculated, which is the parameter best used to evaluate BBB disruption because it takes into account the blood concentrations, which can vary with factors such as body size. Brain/serum ratios were calculated by dividing the brain %Inj/g by serum %Inj/µL and expressed as ul/g.

### 4.8. Protein Extractions from Brain

Frozen brains were homogenized in 1 mL ice-cold detergent-free RIPA buffer (150 mM NaCl, 1M Tris-HCl pH = 8.0, 5 mM MgSO_4_, and Roche Complete Mini protease inhibitor (Sigma)) using the Bead Ruptor 12 (Omni International, Kennesaw, GA, USA) with 1.0 mm zirconia/silica beads (Biospec) at 6 m/s for 30 s. Samples were placed on ice for 5 min, and then the homogenization was repeated. The samples were then partitioned into separate tubes for (a) Aβ extraction, with 720 µL homogenate added to a 2 mL screw-cap tube containing 80 µL buffer (0.1% Triton-X in detergent-free RIPA) and 100 µL zirconia/silica beads; and (b) protein extraction for western blots, with 225 µL homogenate added to a 2 mL screw-cap tube containing 25 µL 10× detergent RIPA buffer (150 mM NaCl, 50 mM Tris-HCl pH = 8.0, 5M MgSO_4_, 10% NP-40, 1% SDS and 5% sodium deoxycholate with protease inhibitors) and 100 µL zirconia/silica beads. These partitioned tubes (a and b) were homogenized for 30 s at 6 m/s, and then left on ice for 15–20 min before the supernatants were aliquoted to fresh tubes and stored in the −80 °C for future use. The remaining detergent-free homogenate not partitioned was spun at 20,000× *g* for 10 min at 4 °C, and then the supernatant was aliquoted into fresh tubes and stored at −80 °C for soluble fractions to measure SAA, which preferentially partitions in the detergent-free fraction. The remaining insoluble pellet was extracted for insoluble Aβ and SAA by adding solubilization buffer (5M guanidine-HCl in PBS with protease inhibitors) and then homogenized on the bead beater at 6 m/s for 30 s. The sample was stored on ice for 5 min, then homogenized two more times. After resting on ice for 5 more minutes, the samples were spun at 20,000× *g* for 20 min at 4 °C before the supernatant was aliquoted and stored at −80 °C. Figure S1 illustrates the extraction scheme.

### 4.9. SAA ELISAs on Serum and Brain

SAA was quantified from serum and brain extracts using the Mouse Serum Amyloid A DuoSet ELISA (R and D Systems, Minneapolis, MN, USA) according to kit instructions. We have validated these kits for SAA measurement in brain and blood using tissue from mice lacking genes for Saa1, Saa2, and Saa3 (donated by Dr. Nancy Webb at the University of Kentucky) and confirmed that signal was absent in the knockouts vs. WT. The kit detects the SAA1 and SAA2 isoforms. Samples were diluted as follows: soluble brain samples were diluted 5-fold (1:4), insoluble brain samples (from guanidine extraction) were diluted 10-fold (1:9), serum from saline groups were diluted 50-fold (1:49), and serum from LPS groups were diluted 1,000,000-fold (1:999,999). Samples were removed from −80 °C and allowed to thaw on ice before centrifugation at 20,000× *g* for 5 min at 4 °C to remove any insoluble debris prior to dilution. All results were normalized to total protein content using the Bradford assay. To calculate tissue/serum ratios of SAA, we divided the concentration of SAA in brain (ng/mg) by the concentration of SAA in serum (ng/mL) and multiplied by 1000 to give final units of µL/mg.

### 4.10. Aβ ELISAs on Brain

Levels of Aβ_1–40_ and Aβ_1–42_ were quantified from brain homogenates using the BAmyloid(40) ELISA WAKO II and BAmyloid(42) ELISA Kit WAKO respectively (Fujifilm Wako Chemicals). Soluble and insoluble brain samples were thawed on ice. Only insoluble brain homogenates from Tg mice were evaluated, since WT mice read below the limit of detection with the required dilutions of guanidine/casein. Prior to all homogenates being centrifuged at 20,000× *g* for 10 min at 4 °C, insoluble samples were diluted 10-fold (1:9) in casein buffer (0.25% *w/v* casein, 5 mM EDTA, pH 8.0) and vortexed. The Aβ_1–40_ kit had limits of detection up to 200 pmol/L. Thus, the soluble samples were diluted 3-fold (1:2) for WT samples or 500-fold (1:499) for Tg2576 samples, while the guanidine/casein samples were diluted 4000-fold (1:3999). The Aβ_1–42_ kit had limits of detection up to 20 pmol/L. Thus, the soluble samples were diluted 3-fold (1:2) for WT samples or 1000-fold (1:999) for Tg samples, while the guanidine/caesin samples were diluted 50,000-fold (1:49,999). All results were normalized to total protein content using the Bradford assay.

### 4.11. Western Blotting

Aliquots of brain homogenates (from RIPA extraction) were thawed on ice and prepared in 4×LDS sample buffer (Invitrogen) and 10× sample reducing agent (Invitrogen) at 1 µg/µL before being heated at 70 °C for 10 min. Cooled samples (10 µg) were run on a NuPAGE 4–12% 26-well 1.0 mm MIDI gel (Invitrogen) with MOPS buffer and transferred onto a nitrocellulose membrane using the iBLOT system (Thermo Fisher, Waltham, MA, USA) at 20 V for 7 min. Membranes were blocked for one hour (TBST, 5% BSA) then incubated with primary antibody overnight (rabbit anti-GFAP; DAKO 1:10,000; Dako, Glostrup, Germany). The following morning, membranes were washed three times with TBST before incubation in secondary anti-rabbit antibody (1:10,000) for 1 h. Then, membranes were washed three times with TBST, incubated in SuperSignal West Pico PLUS Chemiluminescent Substrate (ThermoFisher), and imaged on a ImageQuant LAS 4000 imager (Cytiva, MA, USA). Blots were then quickly dried and probed and imaged with Fast Green FCF (Sigma) according to manufacturer’s instructions.

### 4.12. Cytokine Assay

All cytokines were quantified from brain homogenates using Bio-Plex Pro Mouse Cytokine 23-plex Assay (BioRad, Hercules, CA, USA) according to manufacturer’s instructions. This assay measures: interleukin (IL)-1α, IL-1β, IL-2, IL-3, IL-4, IL-5, IL-6, IL-9, IL-10, IL-12(p40), IL-12(p70), IL-13, IL-17, eotaxin (CCL11), granulocyte colony-stimulating factor (G-CSF), granulocyte-macrophage colony-stimulating factor (GM-CSF), interferon (IFN)-γ, keratinocyte chemoattractant (KC; CXCL1), monocyte chemoattractant protein (MCP)-1 (CCL2), macrophage inflammatory protein (MIP)-1α (CCL3), MIP-1β (CCL4), regulated on activation, normal T cell expressed and secreted (RANTES; CCL5), and tumor necrosis factor (TNF)-α. Soluble brain samples were thawed on ice, vortexed and centrifuged at 20,000× *g* for 5 min at 4 °C prior to diluting 4-fold (1:3) in sample diluent provided in the kit. Matrix diluent was prepared by adding 0.1% Triton X-100 and 1.5% bovine serum albumin (BSA) to brain homogenization buffer. Samples were loaded on the plate in duplicate and normalized to the standard provided. The plate was read on a BioPlex 200 (BioRad). All samples with poor washout were included; this difference in approach is based on SAA levels being greatly affected by blood contamination in the brain whereas cytokines were not. All results were normalized to total protein content using the BCA assay (ThermoFisher).

### 4.13. Statistics

GraphPad Prism 8 was used for all statistical analysis. The effects of treatment and Aβ overexpression on measured outcomes was determined by two-way ANOVA and Tukey’s multiple comparisons test. For statistical comparisons of the differences in means between treatment groups in each genotype, we subtracted the mean value for LPS from the mean value for saline. The *n* was equivalent for all groups. The pooled standard deviation was calculated using Cohen’s method shown below:

$$SDpooled=\sqrt{\frac{SD1^{2}+SD2^{2}}{2}}$$

The difference in means and pooled standard deviations were compared statistically by two-tailed *t*-test. Two-tailed *t-*tests were also used when comparing means of two groups. For Table 2, we used the baseline correction analysis to calculate the change in Sal vs. LPS for each genotype. We then graphed the mean and SEM for each genotype and used an unpaired two-tailed *t*-test to determine significance.

## Figures and Tables

**Table 1:** Results of Two-way ANOVA of all 23 cytokines evaluated. All effects with *p* < 0.05 are bolded and highlighted yellow. *n* = 6 mice/group.

	Cytokines	Genotype Effect	Treatment Effect	Interaction
No Significant Effect	IFN-γ	F (1, 20) = 2.558, *p* = 0.1255	F (1, 20) = 3.503, *p* = 0.0760	F (1, 20) = 1.296, *p* = 0.2684
	IL-9	F (1, 20) = 1.000, *p* = 0.3292	F (1, 20) = 3.524, *p* = 0.0751	F (1, 20) = 3.408, *p* = 0.0797
	TNF-α	F (1, 20) = 4.052, *p* = 0.0578	F (1, 20) = 1.879, *p* = 0.1856	F (1, 20) = 2.960, *p* = 0.1008
Genotype Effect	IL-12 (p70)	**F (1, 20) = 5.880, *p* = 0.0249**	F (1, 20) = 0.05485, *p* = 0.8172	F (1, 20) = 0.6215, *p* = 0.4397
Treatment Effect	Eotaxin/CCL11	F (1, 20) = 0.1027, *p* = 0.7520	**F (1, 20) = 31.34, *p* < 0.0001**	F (1, 20) = 0.2071, *p* = 0.6539
	G-CSF	F (1, 20) = 1.168, *p* = 0.2926	**F (1, 20) = 19.11, *p* = 0.0003**	F (1, 20) = 1.167, *p* = 0.2930
	IL-2	F (1, 20) = 0.06101, *p* = 0.8074	**F (1, 20) = 4.828, *p* = 0.0399**	F (1, 20) = 4.296, *p* = 0.0513
	IL-3	F (1, 20) = 2.263, *p* = 0.1481	**F (1, 20) = 4.828, *p* = 0.0400**	F (1, 20) = 3.040, *p* = 0.0966
	IL-4	F (1, 20) = 2.263, *p* = 0.1481	**F (1, 20) = 4.828, *p* = 0.0400**	F (1, 20) = 3.040, *p* = 0.0966
	IL-5	F (1, 20) = 0.6788, *p* = 0.4197	**F (1, 20) = 5.129, *p* = 0.0348**	F (1, 20) = 1.815, *p* = 0.1929
	IL-13	F (1, 18) = 0.2147, *p* = 0.6487	**F (1, 18) = 4.417, *p* = 0.0499**	F (1, 18) = 0.9062, *p* = 0.3537
	KC/CXCL1	F (1, 20) = 0.9386, *p* = 0.3442	**F (1, 20) = 22.62, *p* = 0.0001**	F (1, 20) = 0.9407, *p* = 0.3437
	MCP-1/CCL2	F (1, 20) = 0.9893, *p* = 0.3318	**F (1, 20) = 10.05, *p* = 0.0048**	F (1, 20) = 1.692, *p* = 0.2082
Interaction Effect	IL-17A	F (1, 20) = 0.03065, *p* = 0.8628	F (1, 20) = 3.941, *p* = 0.0610	**F (1, 20) = 8.585, *p* = 0.0083**
Treatment and Interaction Effect	GM-CSF	F (1, 20) = 1.129, *p* = 0.3006	**F (1, 20) = 5.398, *p* = 0.0308**	**F (1, 20) = 4.516, *p* = 0.0462**
	IL-1α	F (1, 20) = 2.489, *p* = 0.1303	**F (1, 20) = 22.01, *p* = 0.0001**	**F (1, 20) = 4.726, *p* = 0.0419**
	IL-1β	F (1, 20) = 0.01399, *p* = 0.9070	**F (1, 20) = 4.991, *p* = 0.0371**	**F (1, 20) = 7.325, *p* = 0.0136**
	IL-6	F (1, 20) = 2.324, *p* = 0.1431	**F (1, 20) = 12.73, *p* = 0.0019**	**F (1, 20) = 6.331, *p* = 0.0205**
	IL-10	F (1, 20) = 0.2897, *p* = 0.5963	**F (1, 20) = 6.925, *p* = 0.0160**	**F (1, 20) = 5.684, *p* = 0.0271**
Genotype, Treatment and Interaction	IL-12 (p40)	**F (1, 20) = 6.855, *p* = 0.0165**	**F (1, 20) = 30.22, *p* < 0.0001**	**F (1, 20) = 7.793, *p* = 0.0113**
	MIP-1α/CCL3	**F (1, 20) = 35.94, *p* < 0.0001**	**F (1, 20) = 19.37, *p* = 0.0003**	**F (1, 20) = 10.56, *p* = 0.0040**
	MIP-1β/CCL4	**F (1, 20) = 5.705, *p* = 0.0269**	**F (1, 20) = 8.780, *p* = 0.0077**	**F (1, 20) = 7.335, *p* = 0.0135**
	RANTES/CCL5	**F (1, 20) = 11.57, *p* = 0.0028**	**F (1, 20) = 62.89, *p* < 0.0001**	**F (1, 20) = 11.05, *p* = 0.0034**

**Table 2:** Differences in the means of WT and Tg response to Sal and LPS. Table of two-tailed t-test results with the difference between means, SEM, and *p*-values is shown, with all effects *p* < 0.05 highlighted and bolded.

Cytokines	Difference between Means ± SEM, *p* Value
IL-1α	1.399 ± 0.6434, *p* = 0.0548
IL-1β	**1.271 ± 0.4696, *p* = 0.0221**
IL-6	**4.634 ± 1.842, *p* = 0.0306**
IL-12 (p40)	**144.4 ± 51.73, *p* = 0.0191**
IL-10	**7.520 ± 3.154, *p* = 0.0383**
IL-17A	**7.157 ± 2.442, *p* = 0.0150**
GM-CSF	4.942 ± 2.326, *p* = 0.0595
MIP-1α/CCL3	**23.62 ± 7.266, *p* = 0.0087**
MIP-1β/CCL4	**26.97 ± 9.959, *p* = 0.0220**
RANTES/CCL5	**113.4 ± 34.12, *p* = 0.0077**

## Data Availability

The data presented in this study are available on reasonable request from the corresponding author.

## Supplementary Materials

The following supporting information can be downloaded at https://www.mdpi.com/article/10.3390/ijms23042377/s1.
