# Supplementary material for: Amyloid Beta Pathology Exacerbates Weight Loss and Brain Cytokine Responses following Low-Dose Lipopolysaccharide in Aged Female Tg2576 Mice
Source: Int J Mol Sci. 2022 Feb 21;23(4):2377. doi: 10.3390/ijms23042377 (PMC8879430; doi:10.3390/ijms23042377)
Supplement: Supplementary file 1 [file ijms-23-02377-s001.zip › ijms-1608849-supplementary.pdf]

Supporting Information

Amyloid beta pathology exacerbates weight loss and brain cytokine responses following low-dose lipopolysaccharide in aged female Tg2576 mice

Rachel C. Knopp [1,2], Kristen K. Baumann [1], Miranda L. Wilson [1], William A. Banks [1,2], Michelle A. Erickson [1,2]*

[1] Geriatrics Research Education and Clinical Center, Veterans Affairs Puget Sound Health Care System, Seattle, WA, 98108 USA
[2] Division of Gerontology and Geriatric Medicine, Department of Medicine, University of Washington School of Medicine, Seattle, WA, 98195 USA
[*] Correspondence: VA Puget Sound Healthcare System, 1660 S. Columbian Way, S-182 Seattle, WA USA 98108; mericks9@uw.edu; Tel.: 1-206-277-1049

Table S1

Cause of death	WT	Tg2576
Unknown- found dead in cage	1	3
Euthanized- bloating with verified tumors or enlarged spleen	4	3
Euthanized- weight loss, tremors, lethargy without tumors	2	2

Table S1. Death and endpoint criteria in non-survivor mice. Numbers reflect the number of mice in each group, out of 19 total mice in the WT group and 20 in the Tg2576 group.

Figure S1

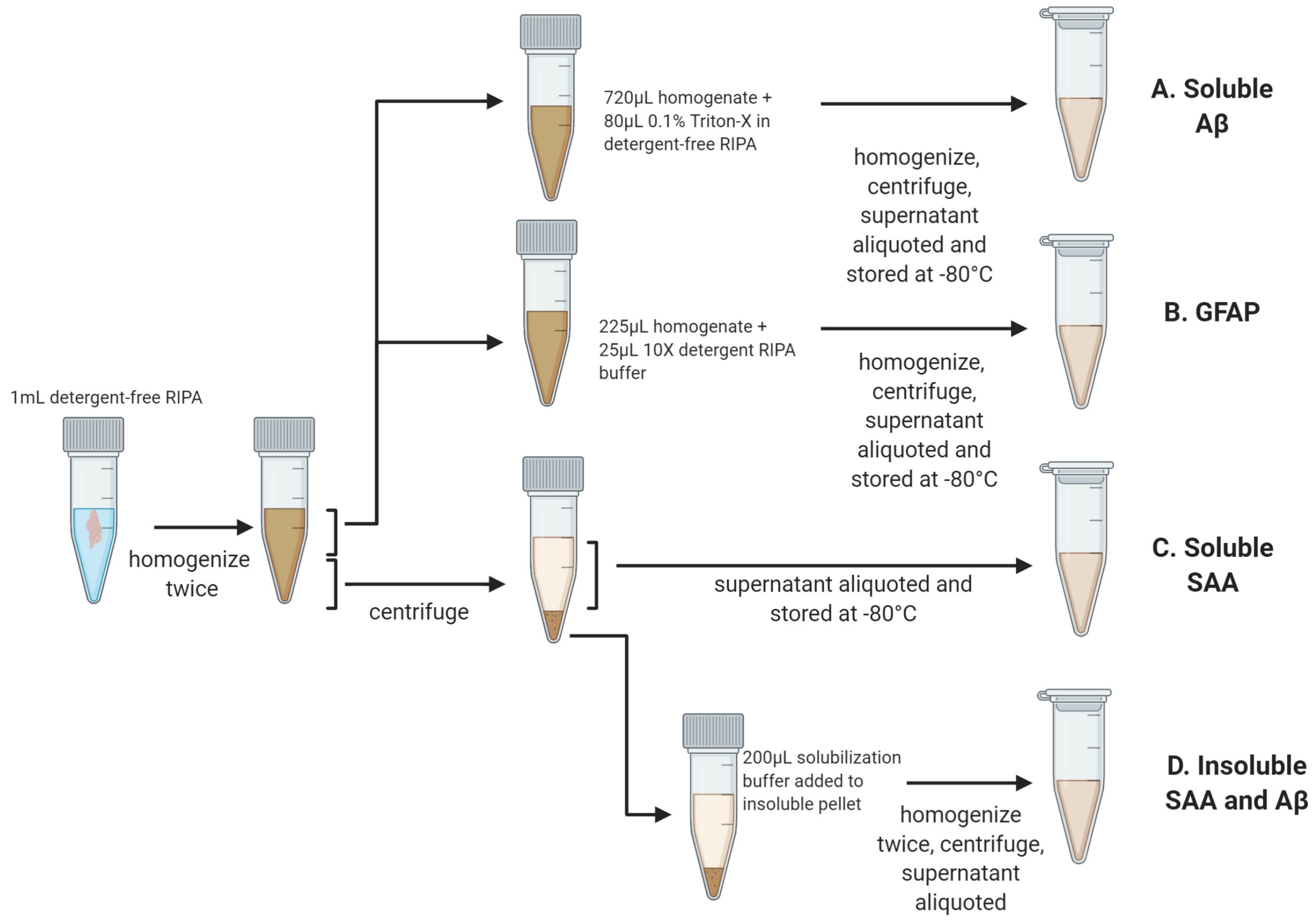

Figure S1. Schematic for extraction of brain tissues for this study. Created with BioRender.com.

Table S2

Cytokine	WT/Sal	WT/LPS	Tg/Sal	Tg/LPS
G-CSF	2	0	2	0
IL-5	0	0	1	0
IL-6	0	1	0	0
IL-13	1	1	3	0

Table 2. Samples with cytokine readings that were either extrapolated from the standard curve (n=12) or were assigned a zero value (n=2).
